# Joint and independent associations of dietary vitamin intake and prevalence of cardiovascular disease in chronic kidney disease subjects: a cross-sectional analysis

**DOI:** 10.3389/fnut.2025.1579313

**Published:** 2025-04-28

**Authors:** Guoqing Wang, Luojun Huang, Wenwen Yue, Jun Feng

**Affiliations:** ^1^Department of Cardiology, Hefei Hospital Affiliated to Anhui Medical University, Hefei, China; ^2^Department of Cardiology, The Fifth Clinical College of Anhui Medical University, Hefei, China

**Keywords:** dietary vitamin intake, cardiovascular disease, prevalence, chronic kidney disease, cross-sectional analysis

## Abstract

**Background:**

Currently, the joint and independent effects of intake of multiple dietary vitamins (including vitamin A, B1, B2, B6, B12, C, D, E, and K) on the prevalence of cardiovascular disease (CVD) in the chronic kidney disease (CKD) population are unclear, so this study was conducted to investigate mainly this point.

**Methods:**

We collected National Health and Nutrition Examination Survey (NHANES) data from 2011 to 2016. We performed weighted multivariate logistic regression models to analyze the association of single dietary vitamins intake with CVD. Additionally, we examined the co-exposure of nine dietary vitamins, defined as their concurrent intake, and evaluated the potential additive or interactive effects of co-exposure of nine dietary vitamins on CVD risk in CKD patients using Bayesian kernel machine regression (BKMR) and weighted quantile sum (WQS) regression.

**Results:**

Finally, 2,203 CKD participants (weighted *n* = 27,120,429) were included, and 622 had CVD, with a CVD prevalence of 28.2%. In the fully adjusted model, by comparing the third tertile with the first tertile, the adjusted OR [T3 vs. T1] for the effect of vitamin B6 on CVD prevalence was 0.67 (95% CI, 0.51–0.89, *p*-value = 0.01), while that of vitamin E was 0.61 (95% CI, 0.42–0.87, *p*-value = 0.01). In the WQS model, the intake of nine dietary vitamins was negatively correlated with CVD prevalence (OR: 0.81, 95% CI: 0.70–0.93, *p*-value = 0.004). In the BKMR model, when the concentration was between the 25th and 75th percentiles, there was an overall negative correlation between the total intake of nine dietary vitamins and CVD prevalence.

**Conclusion:**

High intakes of vitamin B6 and vitamin E were associated with low CVD risk in CKD patients, respectively. Additionally, nine dietary vitamins (vitamins A, B1, B2, B6, B12, C, D, E, and K) co-exposure were inversely correlated with the CVD prevalence in the CKD populations.

## Introduction

Cardiovascular disease (CVD) remains a predominant cause of morbidity and mortality worldwide (1, 2), with a notably higher prevalence among individuals with chronic kidney disease (CKD) (3, 4). Preventing the onset of CVD is therefore of paramount importance within the CKD population. The heightened risk of CVD among patients with CKD stems from a convergence of traditional risk factors, including hypertension and dyslipidemia, alongside non-traditional factors such as chronic inflammation, oxidative stress, and mineral metabolism disturbances (3, 5–7). This increased vulnerability underscores the necessity of systematically examining modifiable factors that may influence cardiovascular outcomes within this cohort. Among these modifiable determinants, dietary vitamin intake has emerged as a focal point due to its potential role in mitigating CVD risk (8, 9).

Vitamins are integral to numerous physiological processes, including antioxidant defense, endothelial function, and lipid metabolism, all of which are critically involved in the pathogenesis of CVD (10–12). Despite these mechanistic advantages, evidence regarding the efficacy of vitamins in reducing CVD risk remains equivocal. Some studies have demonstrated associations between specific vitamins, such as vitamins D, E, and C, and a reduction in cardiovascular risk and mortality within the general population (13–15). Conversely, a study by Desai et al. reported a lack of cardiovascular benefit from vitamin supplementation in the general U.S. population. Furthermore, Fortmann et al. identified no significant impact of folic acid, vitamin C, vitamin A, vitamin D, and/or calcium supplementation on CVD, cancer, or mortality outcomes (16, 17). These inconsistent findings indicate a need for further investigation into the relationship between vitamin intake and disease risk, particularly among populations that may derive specific benefits from vitamin supplementation.

Given the altered metabolism and nutritional challenges inherent to patients with CKD, elucidating the impact of multiple dietary vitamin intakes on CVD prevalence within this population is crucial. Evidence from several studies indicates potential cardiovascular benefits associated with vitamin intake among patients with CKD (8, 18). For instance, Li et al. identified an inverse association between dietary vitamin E intake and CKD prevalence among US adults (OR: 0.86, 95% CI: 0.74–1.00) (19). Similarly, Cheung et al. reported that adequate vitamin K intake may correlate with a reduced risk of CVD mortality in patients with CKD (20). Despite the potential benefit of individual vitamins, real-world clinical settings often involve simultaneous exposure to multiple vitamins. However, the association between multivitamin co-exposure and CVD prevalence in patients with CKD remains inadequately characterized.

This study aims to investigate the independent protective effects of nine dietary vitamins (A, B1, B2, B6, B12, C, D, E, and K) on CVD within CKD populations and to assess the dose–response relationship between multivitamin co-exposure and CVD risk. By examining the combined effects of multivitamin intake, this research endeavors to generate a comprehensive understanding of the role dietary vitamins may play in cardiovascular outcomes among this high-risk group. The findings are expected to inform dietary guidelines and therapeutic interventions aimed at reducing CVD risk in CKD populations, thereby improving their prognosis and quality of life.

## Methods and materials

### Study design and population

This study employed a cross-sectional design, utilizing data from the National Health and Nutrition Examination Survey (NHANES), a nationally representative survey conducted by the Centers for Disease Control and Prevention (CDC). NHANES systematically collects data on various health and nutritional parameters through structured interviews, physical examinations, and laboratory assessments.

**Figure 1:**
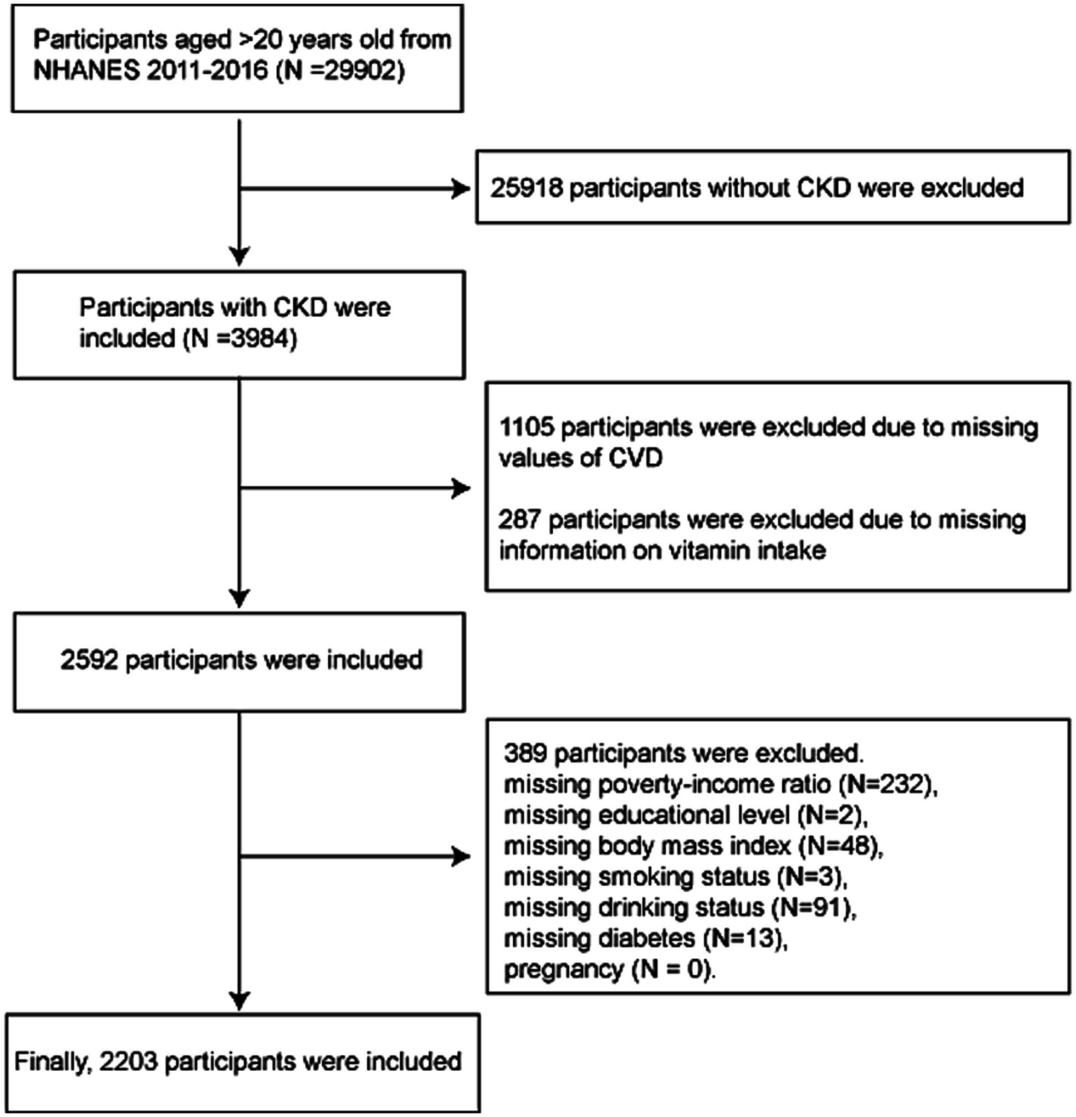
The flow chart. CKD, chronic kidney disease; CVD, cardiovascular disease.

Figure 1 illustrates the participant selection process. Of the 29,902 individuals aged > 20 years from NHANES 2011–2016 (where complete dietary vitamin intake data were available for the three cycles from 2011 to 2016), 25,918 participants were excluded due to the absence of CKD diagnosis. Among the remaining 3,984 CKD participants, exclusions were made for 1,105 individuals with incomplete CVD history and 287 with missing dietary vitamin intake data. An additional 389 participants were excluded due to missing covariate information (including poverty-income ratio, educational level, body mass index, smoking status, drinking status, and diabetes). Consequently, 2,203 participants were finally included in the analysis, comprising 622 individuals with CVD and 1,581 without, yielding a CVD prevalence of 28.2%. Based on KDIGO 2021 guidelines, CKD diagnosis was based on an estimated glomerular filtration rate (eGFR) of < 60 mL/min/1.73 m^2^ or the presence of albuminuria (urinary albumin-to-creatinine ratio, UACR ≥ 30 mg/g) for a period of 3 months or longer (21).

The NHANES protocol received approval from the National Center for Health Statistics (NCHS) Research Ethics Review Board, with informed consent obtained from all participants, thereby negating the need for additional ethical approval for this study.

### Dietary vitamin intake and cardiovascular disease assessment

Dietary vitamin intake was measured through 24-h dietary recall interviews, as part of the NHANES data collection. Following previous methodology (22), the study quantified the intake of specific vitamins, including A, B1, B2, B6, B12, C, D, E, and K. Total vitamin intake was calculated as the sum of self-reported vitamin supplement consumption and dietary intake (23). For analytical purposes, each vitamin intake level was categorized into three tertiles: Tertile 1 (T1), Tertile 2 (T2), and Tertile 3 (T3).

The prevalence of CVD, encompassing angina, coronary heart disease (CHD), congestive heart failure (CHF), heart attack, and stroke, was determined based on self-reported medical history. Participants were considered positive for CVD if they responded affirmatively to the question: “Has a doctor or other health professional ever told you that you had angina, CHD, CHF, heart attack, or stroke?” (24).

### Covariates

Data on the covariates were derived from the NHANES questionnaire, laboratory results, and physical examination records, encompassing age (≤ 65 years/> 65 years), sex (male/female), ethnicity (White/Mexican/Black/Other), marital status (married/other), education level (less than high school graduate/high school graduate or GED/some college or above), poverty-income ratio (< 1.0/≥ 1.0), body mass index (≤ 30 kg/m^2^/> 30 kg/m^2^), smoking status (never/former/current), drinking status (never/former/current), diabetes (No/Yes), hyperlipidemia (No/Yes), and hypertension (No/Yes). Comprehensive data are available at https://www.cdc.gov/nchs/nhanes/.

Diabetes was diagnosed based on one of the following criteria: (1) self-reported diabetes; (2) glycohemoglobin (HbA1c) ≥ 6.5%; (3) fasting glucose (mmol/l) ≥ 7.0; (4) random blood glucose (mmol/l) ≥ 11.1; (5) 2-hour OGTT blood glucose (mmol/l) ≥ 11.1; (6) use of diabetes medication or insulin. Hypertension was diagnosed when any of the following conditions were met: (1) self-reported hypertension; (2) use of antihypertensive medication; (3) systolic blood pressure ≥ 140 mmHg; (4) diastolic blood pressure ≥ 90 mmHg. Hyperlipidemia was diagnosed when one of the following criteria was fulfilled: (1) self-reported hyperlipidemia; (2) use of anti-hyperlipidemic drugs; (3) triglycerides ≥ 150 mg/dL; (4) total cholesterol ≥ 200 mg/dL; (5) high-density lipoprotein cholesterol (HDL-C) < 50 mg/dL in females and < 40 mg/dL in males; (6) low-density lipoprotein cholesterol (LDL-C) ≥ 130 mg/dL.

### Statistical analysis

The statistical analysis accounted for the multistage, stratified sampling, and weighting design of the NHANES database, ensuring the calculation of nationally representative estimates (24). Continuous variables were presented as mean ± standard deviation (SD) or median (interquartile range, IQR), while categorical variables were reported as percentages.

Weighted logistic regression models assessed the associations between single dietary vitamin intake (both continuous and categorical variables: T1, T2, and T3) and the prevalence of CVD among the CKD population. Adjusted odds ratios (ORs) with 95% confidence intervals (CIs) were provided. Additionally, weighted restricted cubic spline (RCS) plots (3 knots) were constructed to illustrate the dose–response relationship between single dietary vitamin intake and CVD prevalence. The *p*-value for nonlinearity was calculated to evaluate the potential linear relationship between single dietary vitamin intake and CVD prevalence.

Furthermore, Bayesian Kernel Machine Regression (BKMR) and Weighted Quantile Sum (WQS) regression were used to assess the potential additive or interactive effects of nine dietary vitamin intakes on CVD prevalence among CKD participants. In the BKMR analysis (model parameters: (1) kernel function: Gaussian; (2) *a priori* distribution for weight calculation: Normal prior), Pearson correlation coefficients were first computed to assess correlations among the nine vitamins. The vitamins were subsequently grouped based on the correlation matrix. The Group Posterior Inclusion Probability (GroupPIP) and Conditional Posterior Inclusion Probability (CondPIP) were then calculated to quantify the probability of each group and individual vitamin being included in the model, reflecting their respective contributions to the overall effect. The global false discovery rate (FDR) was computed to assess the reliability of the associations between co-exposures of nine dietary vitamins and CVD. For WQS regression, the combined effects of nine dietary vitamin intakes on CVD prevalence were evaluated by constructing an index representing the weighted sum of exposure quantiles. Each vitamin intake was ranked by quantiles, and a weighted sum was created, with the weights representing each vitamin’s contribution to overall CVD risk. The WQS index was included as a predictor in a logistic regression model to examine its association with CVD prevalence in CKD populations. The estimated weights further represented the relative importance of each vitamin to the overall effect. To mitigate selection bias from excluding substantial missing data and to ensure result stability, multiple imputation of missing covariate values was performed. The association of co-exposure to the nine dietary vitamins with CVD prevalence was then reassessed using WQS regression and BKMR.

Statistical analysis was conducted using R 4.3.3 and STATA 15 software. All tests were two-tailed, with a *p*-value < 0.05 considered statistically significant.

## Results

### Baseline characteristics of the present study

This study enrolled 2,203 CKD participants aged > 20 years, with a mean (SD) age of 62.05 (16.34) years and 47.30% male. As presented in Table 1, participants with CVD were more likely to be older (> 65 years: 69.29% vs. 42.31%, *p*-value < 0.001), male (53.86% vs. 44.72%, *p*-value < 0.001), and white (53.38% vs. 43.33%, *p*-value < 0.001), compared to those without CVD. They also had a lower marriage rate (45.02% vs. 50.66%, *p*-value = 0.02), a lower proportion of individuals with some college education or higher (40.35% vs. 51.74%, *p*-value < 0.001), higher smoking rates (current smokers: 19.94% vs. 16.51%, *p*-value < 0.001), higher rates of former drinking (37.94% vs. 22.83%, *p*-value < 0.001), and a higher prevalence of diabetes (54.66% vs. 35.67%, *p*-value < 0.001), hyperlipidemia (90.51% vs. 77.99%, *p*-value < 0.001), and hypertension (86.98% vs. 65.72%, *p*-value < 0.001). Additionally, CKD participants with CVD exhibited lower daily vitamin intake levels for vitamins B1, B6, B12, E, and K (*p*-value < 0.05). Only 45.53% of participants met the recommended daily intake (RDI) for vitamin B6, 65.14% for vitamin B12, 8.40% for vitamin E, and 38.40% for vitamin K. These adequacy rates were significantly lower in the CVD group (all *p*-value < 0.05) (Supplementary Table S1).

**Table 1:** Baseline characteristics of the present study.

Variables	Participants, No. (%)			*p*-value
	Total *N* = 2,203	No CVD	CVD	
		*N* = 1,581	*N* = 622	
Age, years				<0.001
≤ 65	1,103 (50.07)	912 (57.69)	191 (30.71)	
> 65	1,100 (49.93)	669 (42.31)	431 (69.29)	
Sex				<0.001
Male	1,042 (47.30)	707 (44.72)	335 (53.86)	
Female	1,161 (52.70)	874 (55.28)	287 (46.14)	
Ethnicity				<0.001
White	1,017 (46.16)	685 (43.33)	332 (53.38)	
Mexican	247 (11.21)	196 (12.40)	51 (8.20)	
Black	524 (23.79)	384 (24.29)	140 (22.51)	
Other	415 (18.84)	316 (19.99)	99 (15.92)	
Marital status				0.02
Married	1,081 (49.07)	801 (50.66)	280 (45.02)	
Other	1,122 (50.93)	780 (49.34)	342 (54.98)	
Education level				<0.001
Less than high school graduate	598 (27.14)	402 (25.43)	196 (31.51)	
High school graduate or general equivalency diploma	536 (24.33)	361 (22.83)	175 (28.14)	
Some college or above	1,069 (48.52)	818 (51.74)	251 (40.35)	
PIR				0.06
< 1.0	547 (24.83)	375 (23.72)	172 (27.65)	
≥ 1.0	1,656 (75.17)	1,206 (76.28)	450 (72.35)	
Body mass index, kg/m2				0.11
≤ 30	1,202 (54.56)	880 (55.66)	322 (51.77)	
> 30	1,001 (45.44)	701 (44.34)	300 (48.23)	
Smoking status				<0.001
Never	1,104 (50.11)	841 (53.19)	263 (42.28)	
Former	714 (32.41)	479 (30.30)	235 (37.78)	
Now	385 (17.48)	261 (16.51)	124 (19.94)	
Drinking status				<0.001
Never	356 (16.16)	261 (16.51)	95 (15.27)	
Former	597 (27.10)	361 (22.83)	236 (37.94)	
Now	1,250 (56.74)	959 (60.66)	291 (46.78)	
Diabetes				<0.001
No	1,299 (58.97)	1,017 (64.33)	282 (45.34)	
Yes	904 (41.03)	564 (35.67)	340 (54.66)	
Hyperlipidemia				<0.001
No	407 (18.47)	348 (22.01)	59 (9.49)	
Yes	1796 (81.53)	1,233 (77.99)	563 (90.51)	
Hypertension				<0.001
No	623 (28.28)	542 (34.28)	81 (13.02)	
Yes	1,580 (71.72)	1,039 (65.72)	541 (86.98)	
Vitamin Types, Median (IQR)				
Vitamin A, μg	446.00 (502.00)	455.00 (505.00)	429.50 (504.75)	0.07
Vitamin B1, mg	1.31 (0.91)	1.33 (0.91)	1.24 (0.90)	<0.01
Vitamin B2, mg	1.67 (1.16)	1.68 (1.18)	1.65 (1.10)	0.08
Vitamin B6, mg	1.60 (1.21)	1.64 (1.29)	1.45 (1.11)	<0.001
Vitamin B12, μg	3.35 (3.65)	3.44 (3.58)	3.11 (3.66)	<0.01
Vitamin C, mg	49.30 (88.3)	50.20 (90.7)	45.30 (83.25)	0.15
Vitamin D, μg	3.10 (4.40)	3.10 (4.40)	3.10 (4.6)	0.27
Vitamin E, mg	6.42 (5.66)	6.74 (5.86)	5.62 (5.08)	<0.001
Vitamin K, ug	62.50 (78.40)	65.70 (82.2)	56.75 (67.22)	<0.001

PIR, poverty-income ratio; IQR, interquartile range (75th quartile minus 25th quartile).

### Association of single dietary vitamin intake with CVD prevalence

**Figure 2:**
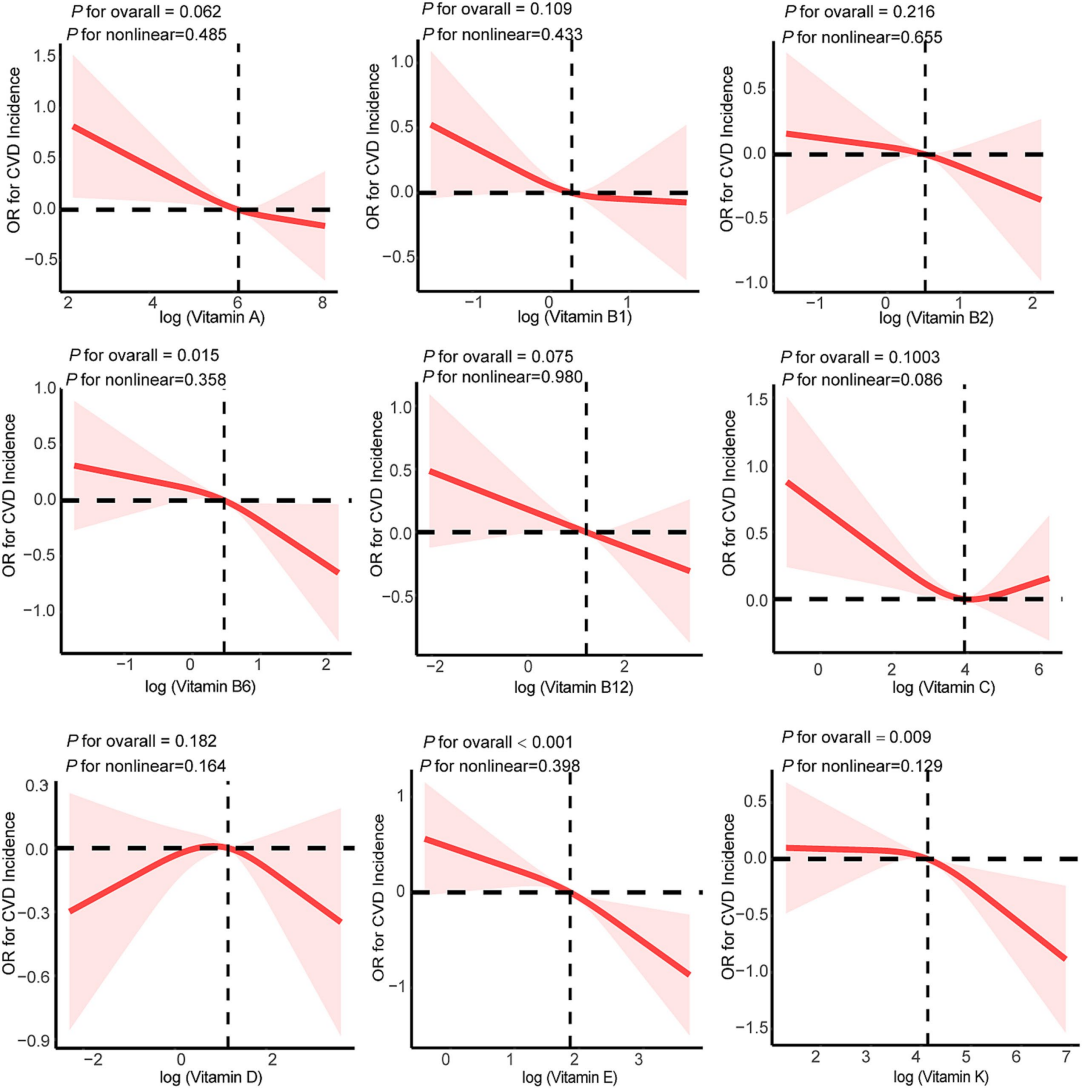
Weighted restricted cubic spline plots for single dietary vitamin intake and CVD prevalence. Age, sex, ethnicity, marital status, poverty-income ratio, education level, body mass index, smoking status, drinking status, diabetes, hyperlipidemia, and hypertension were adjusted. CVD, cardiovascular disease.

Among the 2,203 CKD participants, 622 had CVD, resulting in a CVD prevalence of 28.2%. Table 2 summarizes the associations between nine dietary vitamin intakes and incident CVD events. In the weighted univariate logistic regression model (Model 1), higher intakes of vitamins B6, C, E, and K were inversely associated with CVD prevalence. After adjusting for age, sex, ethnicity, marital status, poverty-income ratio, education level, body mass index, smoking status, drinking status, diabetes, hyperlipidemia, and hypertension (Model 2), high intakes of vitamin B6 and vitamin E were found to be associated with a reduced CVD prevalence. When comparing the third tertile with the first tertile, the adjusted OR [T3 vs. T1] for vitamin B6 on CVD prevalence was 0.67 (95% CI, 0.51–0.89, *p*-value = 0.01), and for vitamin E, it was 0.61 (95% CI, 0.42–0.87, *p*-value = 0.01). Similar results were observed after reanalyzing the data following multiple imputation of missing covariate values (Supplementary Table S2). A linear relationship between vitamin B6, vitamin E, and vitamin K intakes and CVD prevalence was confirmed using RCS regression (*p*-value-values for nonlinearity: *p*-value = 0.358 for vitamin B6, *p*-value = 0.398 for vitamin E, *p*-value = 0.129 for vitamin K) (Figure 2).

**Table 2:** Association of single dietary vitamin intake with CVD prevalence among CKD populations.

Variables	Model 1		Model 2	
	OR 95% CI	*p*-value	OR 95% CI	*p*-value
Tertiles of vitamin A, μg				
Q1	ref		ref	
Q2	0.91 (0.70, 1.18)	0.47	0.86 (0.66, 1.12)	0.26
Q3	0.81 (0.62, 1.07)	0.14	0.83 (0.61, 1.13)	0.23
Tertiles of vitamin B1, mg				
Q1	ref		ref	
Q2	0.92 (0.70, 1.21)	0.55	0.92 (0.67, 1.26)	0.60
Q3	0.89 (0.63, 1.25)	0.49	0.92 (0.64, 1.34)	0.67
Tertiles of vitamin B2, mg				
Q1	ref		ref	
Q2	1.02 (0.76, 1.35)	0.91	1.03 (0.78, 1.37)	0.83
Q3	0.84 (0.64, 1.11)	0.21	0.88 (0.64, 1.21)	0.41
Tertiles of vitamin B6, mg				
Q1	ref		ref	
Q2	0.70 (0.53, 0.93)	0.02	0.76 (0.55, 1.03)	0.08
Q3	0.60 (0.45, 0.79)	<0.001	0.67 (0.51, 0.89)	0.01
Tertiles of vitamin B12, μg				
Q1	ref		ref	
Q2	0.94 (0.73, 1.21)	0.62	1.02 (0.75, 1.38)	0.91
Q3	0.95 (0.71, 1.26)	0.70	0.98 (0.69, 1.39)	0.93
Tertiles of vitamin C, mg				
Q1	ref		ref	
Q2	0.82 (0.66, 1.04)	0.10	0.80 (0.61, 1.05)	0.11
Q3	0.71 (0.56, 0.91)	0.01	0.80 (0.63, 1.02)	0.07
Tertiles of vitamin D, μg				
Q1	ref		ref	
Q2	0.92 (0.67, 1.27)	0.61	0.83 (0.60, 1.16)	0.26
Q3	1.03 (0.76, 1.39)	0.84	0.92 (0.66, 1.29)	0.64
Tertiles of vitamin E, mg				
Q1	ref		ref	
Q2	0.64 (0.47, 0.87)	0.01	0.73 (0.51, 1.04)	0.08
Q3	0.52 (0.38, 0.72)	<0.001	0.61 (0.42, 0.87)	0.01
Tertiles of vitamin K, μg				
Q1	ref		ref	
Q2	0.85 (0.63, 1.13)	0.25	0.91 (0.69, 1.19)	0.47
Q3	0.62 (0.47, 0.81)	<0.001	0.74 (0.54, 1.02)	0.07

Model 1: No variables were adjusted. Model 2: Age, sex, ethnicity, marital status, poverty-income ratio, education level, body mass index, smoking status, drinking status, diabetes, hyperlipidemia, and hypertension were adjusted. OR, odds ratio; CI, confidence interval; CKD, chronic kidney disease; CVD, cardiovascular disease.

### Multi-vitamin exposures and incident CVD risk

**Figure 3:**
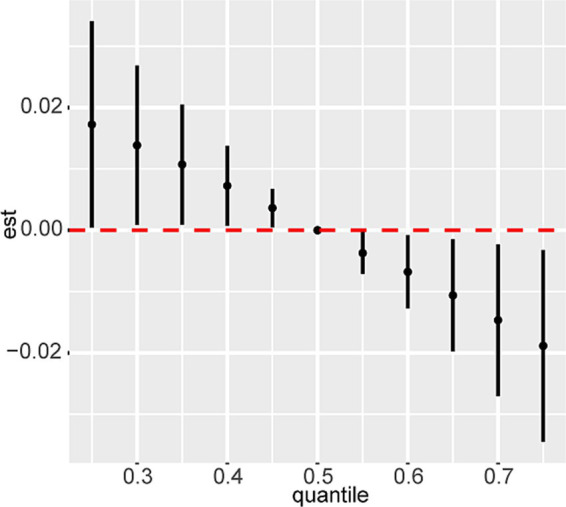
Combined effects of nine dietary vitamins mixtures and CVD prevalence by BKMR analysis. Model was adjusted for age, sex, ethnicity, marital status, poverty-income ratio, education level, body mass index, smoking status, drinking status, diabetes, hyperlipidemia, and hypertension. CVD, cardiovascular disease.

Initially, the BKMR model was employed to investigate the accumulation and potential interactions of nine dietary vitamins. The model revealed a significant negative association between co-exposure to these vitamins and CVD prevalence (Figure 3). Vitamin B2 was found to be strongly correlated with vitamin A (*r* = 0.63), vitamin B1 (*r* = 0.74), vitamin B6 (*r* = 0.65), vitamin B12 (*r* = 0.68), and vitamin D (*r* = 0.60). Additionally, vitamin E and vitamin K exhibited a moderate correlation (*r* = 0.55) (Supplementary Figure S1). Based on these correlations, the nine dietary vitamins were grouped into three categories: group 1 (vitamins A, B1, B2, B6, B12, and D), group 2 (vitamin C), and group 3 (vitamins E and K). Supplementary Figure S2 illustrates the exposure-response trends for different vitamin combinations. When the intake of other vitamins was held at median levels, a stronger association with CVD prevalence was observed for vitamin E and vitamin K. Furthermore, group 3 (vitamins E and K) showed the largest groupPIP, with condPIP values for vitamin E and vitamin K of 0.358 and 0.642, respectively (Supplementary Table S3). These results suggest that vitamin K contributed most to the BKMR model, followed by vitamin E. The global false discovery rate (FDR) was calculated to be < 0.05, indicating that the associations of all screened variables with CVD were robust at the selected PIP threshold (> 0.9).

Subsequently, WQS regression was used to explore the combined effects of co-exposure to nine dietary vitamins on CVD prevalence, given its effectiveness in describing mixtures. The WQS index for the nine vitamins was negatively associated with CVD prevalence (OR: 0.81, 95% CI: 0.70–0.93, *p*-value = 0.004) (Table 3). Among the dietary vitamins, vitamin E (38.86%) and vitamin K (32.61%) were identified as the most influential contributors in the WQS models (Supplementary Table S4 and Supplementary Figure S3).

**Table 3:** The joint effect of nine dietary vitamins on CVD prevalence by WQS model.

Model	OR 95% CI	*p*-value
WQS model	0.81 (0.70, 0.93)	0.004

Model was adjusted for age, sex, ethnicity, marital status, poverty-income ratio, education level, body mass index, smoking status, drinking status, diabetes, hyperlipidemia, and hypertension. WQS, weighted quantile sum; OR, odds ratio; CI, confidence interval; CVD, cardiovascular disease.

### Sensitivity analysis

To address potential selection bias arising from the exclusion of substantial missing data and to enhance the stability of the results, multiple imputation was applied to the missing covariate values. The reanalysis confirmed that co-exposure to the nine dietary vitamins remained negatively correlated with CVD prevalence (WQS model: OR: 0.77, 95% CI: 0.67–0.88, *p*-value < 0.001) (Supplementary Table S5 and Supplementary Figure S4).

## Discussion

This study examined the independent and joint associations between nine dietary vitamins and CVD prevalence in CKD populations, revealing that high intakes of vitamins B6 and E were, respectively, associated with lower CVD prevalence. Moreover, BKMR and WQS regression analyses indicated a negative correlation between the co-exposure to these nine dietary vitamins (A, B1, B2, B6, B12, C, D, E, and K) and low CVD prevalence in CKD populations.

The relationship between dietary vitamins and cardiovascular health has been extensively explored, yet findings remain inconsistent. Wang et al. identified dietary vitamins E and C as significant predictors of CVD risk in adults (25), while Flynn et al. concluded that vitamin E does not prevent cardiovascular events in high-risk patients (26). Furthermore, Tang et al. reported an inverse association between vitamin A intake and cardiometabolic multimorbidity risk (HR 0.66, 95% CI 0.54–0.81) (27). Matos et al. found that vitamin A helped reduce oxidative stress, potentially lowering the risk of postoperative complications in cardiac surgery patients (28). In contrast, Huk et al. demonstrated that excessive vitamin A intake promoted heart valve calcification in mice (29). These conflicting results have sparked further investigation into whether vitamin intake can enhance cardiovascular health. In CKD populations, where vitamin intake and metabolism can vary significantly, vitamin deficiencies are common, and supplementation has been shown to reduce atherosclerotic arterial stiffness and lower CVD risk (30–32). The impact of individual vitamins on cardiovascular health in patients with CKD remains critical (32–35). Consistent with earlier studies, the present research analyzed the relationship between the intake of nine dietary vitamins (A, B1, B2, B6, B12, C, D, E, and K) and CVD prevalence in CKD populations. Results indicated that higher intakes of vitamin B6 (OR: 0.67, 95% CI: 0.51–0.89) and vitamin E (OR: 0.61, 95% CI: 0.42–0.87) were associated with a reduced likelihood of CVD prevalence. Furthermore, re-analysis after multiple imputation of missing data revealed a negative dose–response relationship between vitamin K intake and CVD prevalence (OR: 0.71, 95% CI: 0.53–0.95), which aligned with the RCS results for vitamin K presented in Figure 2. The observed difference in the vitamin K-CVD relationship before and after imputation likely stems from the reduced sample size following the exclusion of missing data.

Vitamin B6 and vitamin E were inversely associated with the prevalence of CVD in patients with CKD, likely due to renal metabolism, inflammatory factors, endothelial dysfunction, and oxidative stress. Vitamin B6 (pyridoxine), a cofactor in homocysteine metabolism, plays a crucial role in regulating homocysteine levels, which are elevated in CKD due to impaired renal clearance. Elevated homocysteine promotes endothelial dysfunction and thrombosis (36), while vitamin B6 deficiency exacerbates hyperhomocysteinemia, further increasing CVD risk. Additionally, vitamin B6 modulates immune responses by reducing pro-inflammatory cytokines (e.g., IL-6, TNF-*α*), which are elevated in CKD and contribute to atherosclerosis (34). Vitamin E (α-tocopherol) counteracts lipid peroxidation and reactive oxygen species (ROS), both of which are elevated in CKD due to uremic toxins (37), likely explaining its protective role in CVD within this cohort. Furthermore, vitamin E enhances nitric oxide bioavailability, improving endothelial function, a key factor in CKD-related CVD (32).

The relationship between multivitamin co-exposure and CVD prevalence in CKD remains underexplored. This study employed advanced statistical methods, including BKMR and WQS regression, revealing that co-exposure to nine dietary vitamins was negatively associated with low CVD prevalence in CKD populations (OR: 0.81, 95% CI: 0.70–0.93). These methods effectively captured the complex interactions and joint effects of multiple vitamins, enabling an explanation of potential synergistic or antagonistic effects, which could clarify inconsistencies in earlier studies focused on individual vitamins. The biological mechanisms linking vitamins to CVD risk in patients with CKD are multifaceted. For example, vitamin E mitigates oxidative stress (37), vitamin K supplementation may prevent or reverse vascular calcification (38), and vitamin B6 reduces CVD risk by lowering homocysteine levels (36). These findings underscore the importance of considering the combined effects of vitamins rather than isolating their individual contributions.

The National Institutes of Health (NIH) recommends a vitamin B6 RDI of 1.3–1.7 mg/day and a vitamin E RDI of 15 mg/day for healthy adults. While this study did not establish absolute intake thresholds, tertile-based analysis indicates that exceeding the general RDI may offer protection for patients with CKD. The median vitamin B6 intake in the non-CVD group was > 1.64 mg, and the median vitamin E intake was > 6.74 mg, suggesting that the benefit may be lower than the general RDI due to metabolic alterations in CKD. Both WQS and BKMR analyses revealed that a balanced intake of dietary vitamins (A, B1, B2, B6, B12, C, D, E, and K) was associated with a 19% reduction in CVD risk (WQS OR 0.81, 95% CI 0.70–0.93), with vitamin E (38.9% weight) and vitamin K (32.6%) playing a prominent protective role. Although specific RDIs for patients with CKD remain undefined, our findings suggest that vitamin B6 intake > 1.64 mg/day and vitamin E intake > 6.74 mg/day, along with a balanced multivitamin, may reduce CVD risk. Future studies should determine the optimal intake thresholds for this population.

Notably, only 45.53% of participants met the RDI for vitamin B6, 8.40% for vitamin E, and 38.40% for vitamin K, with these adequacy rates significantly lower in the CVD group. In contrast, vitamin B2 showed a higher adequacy rate (78.30% vs. 77.67%), with no significant differences between the CVD and non-CVD groups (*p*-value > 0.05). This finding aligns with prior reports of micronutrient deficiencies in CKD due to dietary restrictions, altered metabolism, and uremic toxin accumulation (8). Although CKD-specific RDIs have not yet been defined, the protective associations observed for vitamin B6 (> 1.64 mg/day) and vitamin E (> 6.74 mg/day) fell below general population RDIs but exceeded the median intakes in our cohort, indicating that patients with CKD may require individualized intake thresholds.

This study carries significant clinical implications for managing CVD risk in patients with CKD. The findings suggest that incorporating a dietary regimen containing nine essential vitamins may help reduce CVD risk. This approach could inform dietary recommendations for patients with CKD, emphasizing a balanced vitamin intake over high doses of individual vitamins. However, while the results are promising, they should be interpreted with caution. First, the cross-sectional design of NHANES data limits the ability to establish causality. Second, unmeasured confounding factors may have influenced the outcomes, despite efforts to control for known confounders. Additionally, due to sample size limitations, the study could not examine different CKD stages, and further research is needed in this area. Future studies should explore the relationship between the intake of these nine dietary vitamins and CVD prevalence across various CKD stages. Moreover, the analysis did not account for vitamin supplement use or the effects of CKD-MBD on vitamin D/K metabolism. Additionally, the duration of CKD and CVD in the participants was not considered, as data limitations restricted this assessment. Finally, dietary intake was assessed through 24-h dietary recall, which may be subject to recall bias and does not reflect long-term dietary patterns.

## Conclusion

In conclusion, moderate increases in dietary vitamin B6 and E intake may reduce CVD risk in CKD populations, with a multivitamin synergy emphasizing the importance of balanced nutrition. Future prospective studies should aim to identify optimal intake thresholds for specific vitamins and evaluate the potential risks of excessive vitamin intake.

## Data Availability

The raw data supporting the conclusions of this article will be made available by the authors, without undue reservation.

## References

[1] Arnett DK Blumenthal RS Albert MA Buroker AB Goldberger ZD Hahn EJ. 2019 ACC/AHA guideline on the primary prevention of cardiovascular disease: executive summary: a report of the American College of Cardiology/American Heart Association task force on clinical practice guidelines. J Am Coll Cardiol. (2019) 74:1376–414. doi: 10.1016/j.jacc.2019.03.009, PMID: 30894319 PMC8344373

[2] Minhas AMK Al-Kindi S Van Spall HGC Abramov D. Comparing cardiovascular mortality estimates from global burden of disease and from the Centers for Disease Control and Prevention wide-ranging online data for epidemiological research. Circ Cardiovasc Qual Outcomes. (2025) 4:e011459. doi: 10.1161/CIRCOUTCOMES.124.011459 40184153

[3] Rahman M Xie D Feldman HI Go AS He J Kusek JW. Association between chronic kidney disease progression and cardiovascular disease: results from the CRIC study. Am J Nephrol. (2014) 40:399–407. doi: 10.1159/000368915, PMID: 25401485 PMC4275411

[4] Hiyamuta H Yamada S Taniguchi M Nakano T Tsuruya K Kitazono T. Causes of death in patients undergoing maintenance hemodialysis in Japan: 10-year outcomes of the Q-cohort study. Clin Exp Nephrol. (2021) 25:1121–30. doi: 10.1007/s10157-021-02089-6, PMID: 34100165

[5] Levey A Atkins R Coresh J Cohen E Collins A Eckardt K-U. Chronic kidney disease as a global public health problem: approaches and initiatives–a position statement from kidney disease improving global outcomes. Kidney Int. (2007) 72:247–59. doi: 10.1038/sj.ki.5002343, PMID: 17568785

[6] Zoccali C. Traditional and emerging cardiovascular and renal risk factors: an epidemiologic perspective. Kidney Int. (2006) 70:26–33. doi: 10.1038/sj.ki.5000417 16723985

[7] Lee DY Moon JS Jung I Chung SM Park SY Yu JH. Risk acceleration by gout on major adverse cardiovascular events and all-cause death in patients with diabetes and chronic kidney disease. Diabetes Obes Metab. (2025) 27:1554–63. doi: 10.1111/dom.16165, PMID: 39748223

[8] Chazot C Steiber A Kopple JD. Vitamin needs and treatment for chronic kidney disease patients. J Ren Nutr. (2023) 33:S21–9. doi: 10.1053/j.jrn.2022.09.008, PMID: 36182060

[9] Trugilho L Alvarenga L Cardozo L Paiva B Brito J Barboza I. Effects of Tocotrienol on cardiovascular risk markers in patients with chronic kidney disease: a randomized controlled trial. J Nutr Metab. (2025) 2025:8482883. doi: 10.1155/jnme/8482883, PMID: 39840146 PMC11745556

[10] Chung E Zhang D Gonzalez Porras M Hsu CG. TREM2 as a regulator of obesity-induced cardiac remodeling: mechanisms and therapeutic insights. Am J Physiol Heart Circ Physiol. (2025) 28:2025. doi: 10.1152/ajpheart.00075.2025, PMID: 40152357 PMC12175171

[11] Zisis M Chondrogianni ME Androutsakos T Rantos I Oikonomou E Chatzigeorgiou A. Linking cardiovascular disease and metabolic dysfunction-associated Steatotic liver disease (MASLD): the role of Cardiometabolic drugs in MASLD treatment. Biomol Ther. (2025) 15:324. doi: 10.3390/biom15030324, PMID: 40149860 PMC11940321

[12] Czlapka-Matyasik M Wadolowska L Gut P Gramza-Michałowska A. Changes in oxidative stress, inflammatory markers, and lipid profile after a 6-week high-antioxidant-capacity dietary intervention in CVD patients. Nutrients. (2025) 17:806. doi: 10.3390/nu17050806, PMID: 40077675 PMC11902212

[13] Zhao S Cao Y Liu H Liu A. Joint and independent associations of dietary antioxidant intakes with all-cause and cardiovascular mortality among patients with hypertension: a population-based cohort study. Nutr J. (2025) 24:14. doi: 10.1186/s12937-024-01062-9, PMID: 39856716 PMC11761209

[14] Jiang M Zhang C. Higher dietary vegetable and fruit intake along with their biomarkers is inversely associated with all-cause mortality among cancer survivors. Nutr Res. (2025) 135:141–57. doi: 10.1016/j.nutres.2025.02.001 40056790

[15] Fang X Zhang J Zhang Z Ye D. Association between vitamin D and mortality risk in gout patients. J Public Health. (2025) 9:fdaf010. doi: 10.1093/pubmed/fdaf010, PMID: 40057967

[16] Fortmann SP Burda BU Senger CA Lin JS Beil TL O'Connor E. U.S. preventive services task force evidence syntheses, formerly systematic evidence reviews. Vitamin, mineral, and multivitamin supplements for the primary prevention of cardiovascular disease and Cancer: A systematic evidence review for the US preventive services task force. Rockville, MD: Agency for Healthcare Research and Quality (2013). 24308073

[17] Desai CK Huang J Lokhandwala A Fernandez A Riaz IB Alpert JS. The role of vitamin supplementation in the prevention of cardiovascular disease events. Clin Cardiol. (2014) 37:576–81. doi: 10.1002/clc.22299, PMID: 24863141 PMC6649521

[18] Bhan I Hewison M Thadhani R. Dietary vitamin D intake in advanced CKD/ESRD. Semin Dial. (2010) 23:407–10. doi: 10.1111/j.1525-139X.2010.00751.x, PMID: 20701720

[19] Li J Liu Z Pu Y Dai H Peng F. Association between dietary vitamin E intake and chronic kidney disease events in US adults: a cross-sectional study from NHANES 2009-2016. Clin Kidney J. (2023) 16:2559–66. doi: 10.1093/ckj/sfad162, PMID: 38046017 PMC10689171

[20] Cheung CL Sahni S Cheung BM Sing CW Wong IC. Vitamin K intake and mortality in people with chronic kidney disease from NHANES III. Clin Nutr. (2015) 34:235–40. doi: 10.1016/j.clnu.2014.03.011, PMID: 24745600

[21] Rovin BH Adler SG Barratt J Bridoux F Burdge KA Chan TM. Executive summary of the KDIGO 2021 guideline for the Management of Glomerular Diseases. Kidney Int. (2021) 100:753–79. doi: 10.1016/j.kint.2021.05.015, PMID: 34556300

[22] Liang F Lu M Zhou Y. Associations between single and multiple dietary vitamins and the risk of periodontitis: results from NHANES 2009-2014. Front Nutr. (2024) 11:1347712. doi: 10.3389/fnut.2024.1347712, PMID: 38650639 PMC11033469

[23] Shen R Hua J Guo X Yu L Qiu M Ma L. The mediating role of depression in the association between socioeconomic status and cardiovascular disease: a nationwide cross-sectional study from NHANES 2005-2018. J Affect Disord. (2024) 366:466–73. doi: 10.1016/j.jad.2024.08.145 39187190

[24] Grande G Ljungman PL Eneroth K Bellander T Rizzuto D. Association between cardiovascular disease and long-term exposure to air pollution with the risk of dementia. JAMA Neurol. (2020) 77:801–9. doi: 10.1001/jamaneurol.2019.4914, PMID: 32227140 PMC7105952

[25] Wang Y Han L Ling S Sha Y Sun H. Dietary intake of potassium, vitamin E, and vitamin C emerges as the most significant predictors of cardiovascular disease risk in adults. Medicine. (2024) 103:e39180. doi: 10.1097/MD.0000000000039180, PMID: 39121250 PMC11315499

[26] Flynn T Stevermer JJ. Vitamin E does not prevent cardiovascular events in high-risk patients. J Fam Pract. (2000) 49:372–3. 10778845

[27] Tang Y Xiao Y Yang F Gao X Zhu X Qiao G. Association between dietary vitamin a intake and risk of cardiometabolic multimorbidity. Sci Rep. (2024) 14:16656. doi: 10.1038/s41598-024-67723-1, PMID: 39030396 PMC11271594

[28] Matos A Souza G Moreira V Luna M Ramalho A. Vitamin a supplementation according to zinc status on oxidative stress levels in cardiac surgery patients. Nutr Hosp. (2018) 35:767–73. doi: 10.20960/nh.1666, PMID: 30070862

[29] Huk DJ Hammond HL Kegechika H Lincoln J. Increased dietary intake of vitamin a promotes aortic valve calcification in vivo. Arterioscler Thromb Vasc Biol. (2013) 33:285–93. doi: 10.1161/ATVBAHA.112.300388, PMID: 23202364 PMC3557503

[30] Shing CM Fassett RG Peake JM Coombes JS. Effect of tocopherol on atherosclerosis, vascular function, and inflammation in apolipoprotein E knockout mice with subtotal nephrectomy. Cardiovasc Ther. (2014) 32:270–5. doi: 10.1111/1755-5922.12096, PMID: 25307205

[31] Galli F Bonomini M Bartolini D Zatini L Reboldi G Marcantonini G. Vitamin E (alpha-tocopherol) metabolism and nutrition in chronic kidney disease. Antioxidants. (2022) 11:989. doi: 10.3390/antiox11050989 35624853 PMC9137556

[32] Gluba-Brzózka A Franczyk B Ciałkowska-Rysz A Olszewski R Rysz J. Impact of vitamin D on the cardiovascular system in advanced chronic kidney disease (CKD) and Dialysis patients. Nutrients. (2018) 10:709. doi: 10.3390/nu10060709, PMID: 29865146 PMC6024710

[33] Gallieni M Fusaro M. Vitamin K and cardiovascular calcification in CKD: is patient supplementation on the horizon? Kidney Int. (2014) 86:232–4. doi: 10.1038/ki.2014.24, PMID: 25079019

[34] Li L Zhao J. Association of serum 25-hydroxyvitamin D with cardiovascular and all-cause mortality in patients with chronic kidney disease: NHANES 2007–2018 results. Clinics. (2024) 79:100437. doi: 10.1016/j.clinsp.2024.100437, PMID: 38996723 PMC11296000

[35] Zhao S Chen X Wan Z Geng T Lu Q Yu H. Associations of serum 25-hydroxyvitamin D and vitamin D receptor polymorphisms with risks of cardiovascular disease and mortality among patients with chronic kidney disease: a prospective study. Am J Clin Nutr. (2024) 119:1397–404. doi: 10.1016/j.ajcnut.2024.04.001, PMID: 38608754

[36] Granieri M Bellisarii FI De Caterina R. Group B vitamins as new variables related to the cardiovascular risk. Ital Heart J Suppl. (2005) 6:1–16. 15776726

[37] Bhatti FU Mehmood A Latief N Zahra S Cho H Khan SN. Vitamin E protects rat mesenchymal stem cells against hydrogen peroxide-induced oxidative stress in vitro and improves their therapeutic potential in surgically-induced rat model of osteoarthritis. Osteoarthr Cartil. (2017) 25:321–31. doi: 10.1016/j.joca.2016.09.014, PMID: 27693502

[38] Neofytou IE Stamou A Demopoulos A Roumeliotis S Zebekakis P Liakopoulos V. Vitamin K for vascular calcification in kidney patients: still alive and kicking, but still a lot to learn. Nutrients. (2024) 16:1798. doi: 10.3390/nu16121798, PMID: 38931153 PMC11206649

