# Supplementary material for: Joint and independent associations of dietary vitamin intake and prevalence of cardiovascular disease in chronic kidney disease subjects: a cross-sectional analysis
Source: Front Nutr. 2025 Apr 28;12:1579313. doi: 10.3389/fnut.2025.1579313 (PMC12066483; doi:10.3389/fnut.2025.1579313)
Supplement: Supplementary file 1 [file Table_1.DOCX]

**Table S1** The percentage of vitamin adequacy in CKD populations.

| Variables | RDI Threshold | Participants, No. (%) | | | *P*-value |
| --- | --- | --- | --- | --- | --- |
|  |  | Overall Adequacy (%) | No CVD Group (%) | CVD Group (%) |  |
|  |  |  | N=1581 | N=622 |  |
| Vitamin A, μg | >700 ug | 601(27.28) | 442(27.96) | 159(25.56) | 0.28 |
| Vitamin B1, mg | >1.1 mg | 1376(62.46) | 998(63.12) | 378(60.77) | 0.33 |
| Vitamin B2, mg | >1.1 mg | 1715(77.85) | 1228(77.67) | 487(78.30) | 0.79 |
| Vitamin B6, mg | >1.7 mg | 1003(45.53) | 752(47.56) | 251(40.35) | <0.01 |
| Vitamin B12, μg | >2.4 ug | 1435(65.14) | 1052(66.54) | 383(61.58) | 0.03 |
| Vitamin C, mg | >75 mg | 821(37.27) | 593(37.51) | 228(36.66) | 0.75 |
| Vitamin D, μg | > 10 ug | 191(8.67) | 139(8.79) | 52(8.36) | 0.81 |
| Vitamin E, mg | >15 mg | 185(8.40) | 152(9.61) | 33(5.31) | <0.01 |
| Vitamin K, ug | >80 ug | 846(38.40) | 647(40.92) | 199(31.99) | <0.001 |

Abbreviations: RDI, recommended dietary intake; CKD, chronic kidney disease; CVD, cardiovascular disease.

**Table S2.** Association of single dietary vitamin intake with CVD prevalence among 2592 CKD populations.

| **Variables** | Model 1 | | Model 2 | |
| --- | --- | --- | --- | --- |
|  | OR 95%CI | P-value | OR 95%CI | P-value |
| Tertiles of vitamin A, μg | |  |  |  |
| T1 | Ref. |  | Ref. |  |
| T2 | 0.96(0.74,1.25) | 0.78 | 0.91(0.70,1.19) | 0.48 |
| T3 | 0.82(0.62,1.07) | 0.14 | 0.81(0.60,1.08) | 0.14 |
| P for trend |  | 0.14 |  | 0.14 |
| Tertiles of vitamin B1, mg | |  |  |  |
| T1 | Ref. |  | Ref. |  |
| T2 | 0.94(0.72,1.25) | 0.68 | 0.93(0.69,1.25) | 0.62 |
| T3 | 0.90(0.66,1.24) | 0.51 | 0.91(0.65,1.29) | 0.60 |
| P for trend |  | 0.51 |  | 0.6 |
| Tertiles of vitamin B2, mg | |  |  |  |
| T1 | Ref. |  | Ref. |  |
| T2 | 1.13(0.86,1.49) | 0.38 | 1.13(0.87,1.48) | 0.35 |
| T3 | 0.88(0.68,1.13) | 0.30 | 0.89(0.66,1.18) | 0.40 |
| P for trend |  | 0.26 |  | 0.37 |
| Tertiles of vitamin B6, mg | |  |  |  |
| T1 | Ref. |  | Ref. |  |
| T2 | 0.739(0.576,0.948) | 0.018 | 0.801(0.608,1.054) | 0.110 |
| T3 | 0.621(0.474,0.813) | <0.001 | 0.689(0.518,0.916) | 0.012 |
| P for trend |  | <0.001 |  | 0.011 |
| Tertiles of vitamin B12, μg | |  |  |  |
| T1 | Ref. |  | Ref. |  |
| T2 | 0.922(0.715,1.190) | 0.525 | 1.002(0.744,1.350) | 0.989 |
| T3 | 0.973(0.751,1.261) | 0.833 | 1.013(0.745,1.377) | 0.933 |
| P for trend |  | 0.852 |  | 0.933 |
| Tertiles of vitamin C, mg | |  |  |  |
| T1 | ref |  | ref |  |
| T2 | 0.84(0.65,1.08) | 0.16 | 0.84(0.63,1.14) | 0.26 |
| T3 | 0.75(0.57,0.98) | 0.03 | 0.85(0.63,1.13) | 0.25 |
| P for trend |  | 0.03 |  | 0.25 |
| Tertiles of vitamin D, μg | |  |  |  |
| T1 | Ref. |  | Ref. |  |
| T2 | 0.99(0.72,1.36) | 0.93 | 0.91(0.65,1.27) | 0.58 |
| T3 | 1.07(0.78,1.45) | 0.67 | 0.94(0.69,1.28) | 0.68 |
| P for trend |  | 0.67 |  | 0.69 |
| Tertiles of vitamin E, mg | |  |  |  |
| T1 | ref |  | ref |  |
| T2 | 0.63(0.47,0.85) | 0.003 | 0.70(0.50,0.98) | 0.04 |
| T3 | 0.50(0.37,0.67) | <0.001 | 0.56(0.40,0.78) | 0.001 |
| P for trend |  | <0.001 |  | <0.001 |
| Tertiles of vitamin K, μg | |  |  |  |
| T1 | Ref. |  | Ref. |  |
| T2 | 0.84(0.63,1.11) | 0.22 | 0.87(0.66,1.14) | 0.30 |
| T3 | 0.59(0.46,0.77) | <0.001 | 0.71(0.53,0.95) | 0.02 |
| P for trend |  | <0.001 |  | 0.02 |

Model 1: No variables were adjusted. Model 2: Age, sex, ethnicity, marital status, poverty-income ratio, education level, body mass index, smoking status, drinking status, diabetes, hyperlipidemia, and hypertension were adjusted. Abbreviations: OR, odds ratio; CI, confidence interval; CKD, chronic kidney disease; CVD, cardiovascular disease.

**Table S3 GroupPIP and CondPIP of nine dietary vitamins for BKMR model.**

| **Variables** | group | groupPIP | condPIP | Bayesian FDR |
| --- | --- | --- | --- | --- |
| Vitamin A, μg | 1 | 0.662 | 0.178 | <0.05 |
| Vitamin B1, mg | 1 | 0.662 | 0.136 |  |
| Vitamin B2, mg | 1 | 0.662 | 0.148 |  |
| Vitamin B6, mg | 1 | 0.662 | 0.227 |  |
| Vitamin B12, μg | 1 | 0.662 | 0.149 |  |
| Vitamin C, mg | 2 | 0.606 | 1.000 |  |
| Vitamin D, μg | 1 | 0.662 | 0.162 |  |
| Vitamin E, mg | 3 | 0.877 | 0.358 |  |
| Vitamin K, μg | 3 | 0.877 | 0.642 |  |

**Table S4 mean weight of nine dietary vitamins for WQS model.**

| **Variables** | mean weight |
| --- | --- |
| Vitamin B2, mg | 0.003137 |
| Vitamin D, μg | 0.004259 |
| Vitamin B1, mg | 0.004413 |
| Vitamin C, mg | 0.011179 |
| Vitamin A, μg | 0.019479 |
| Vitamin B12, μg | 0.100535 |
| Vitamin B6, mg | 0.142276 |
| Vitamin K, μg | 0.326149 |
| Vitamin E, mg | 0.388572 |


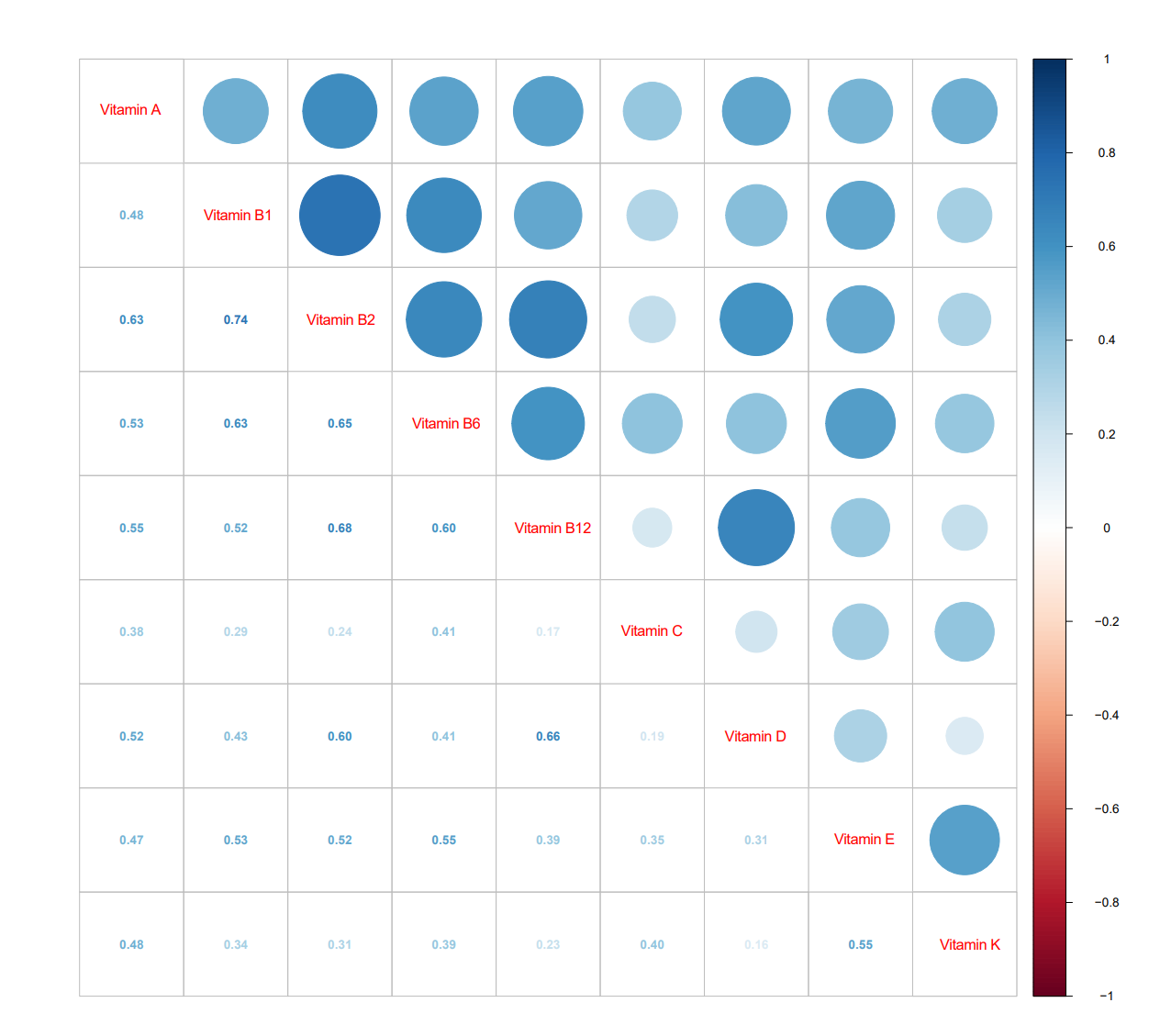


**Figure S1.** The correlation of the intake of nine dietary vitamins


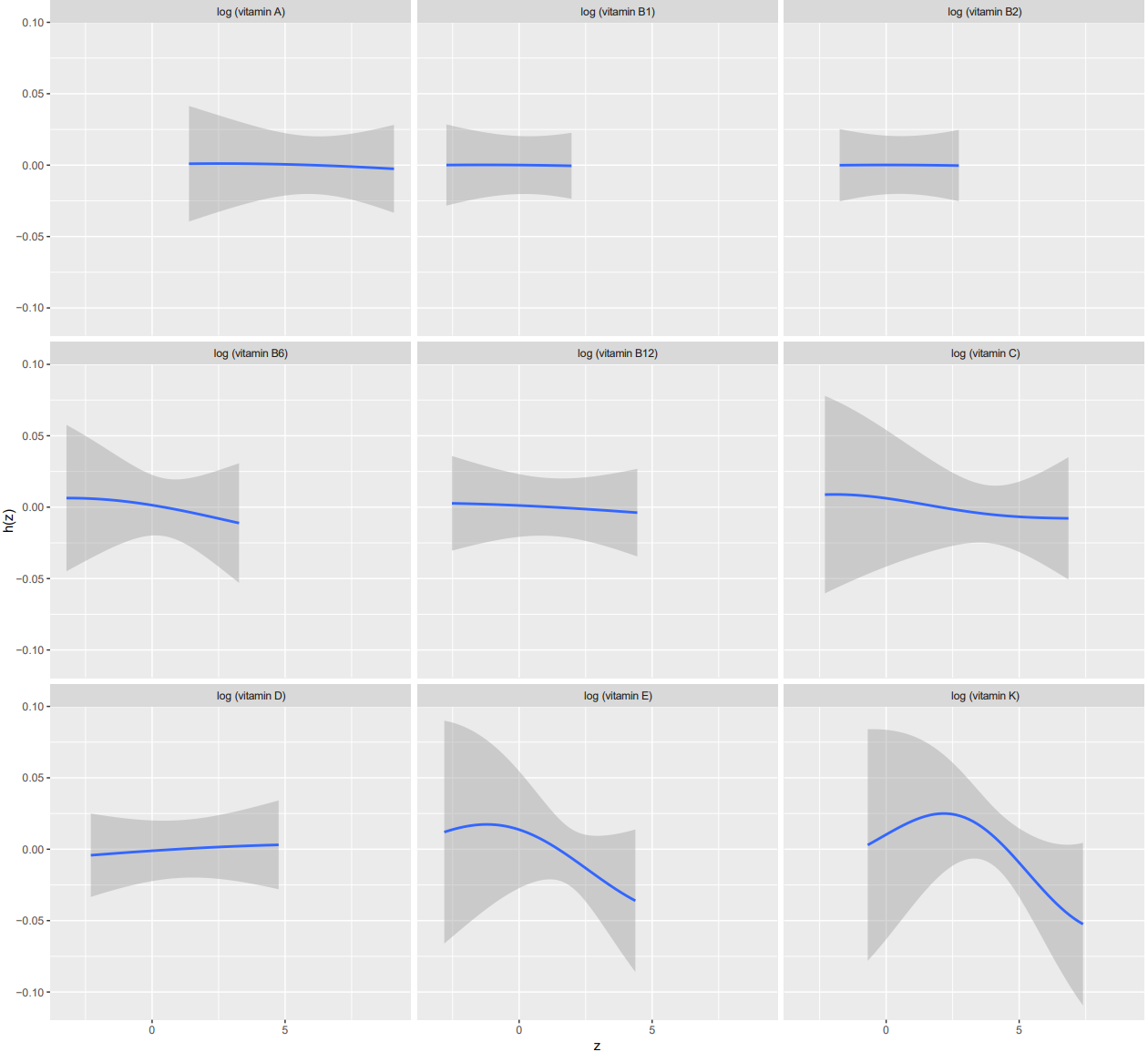


**Figure S2.** Exposure-response functions for each dietary vitamin intake with the other dietary vitamin intake fixed at the median.


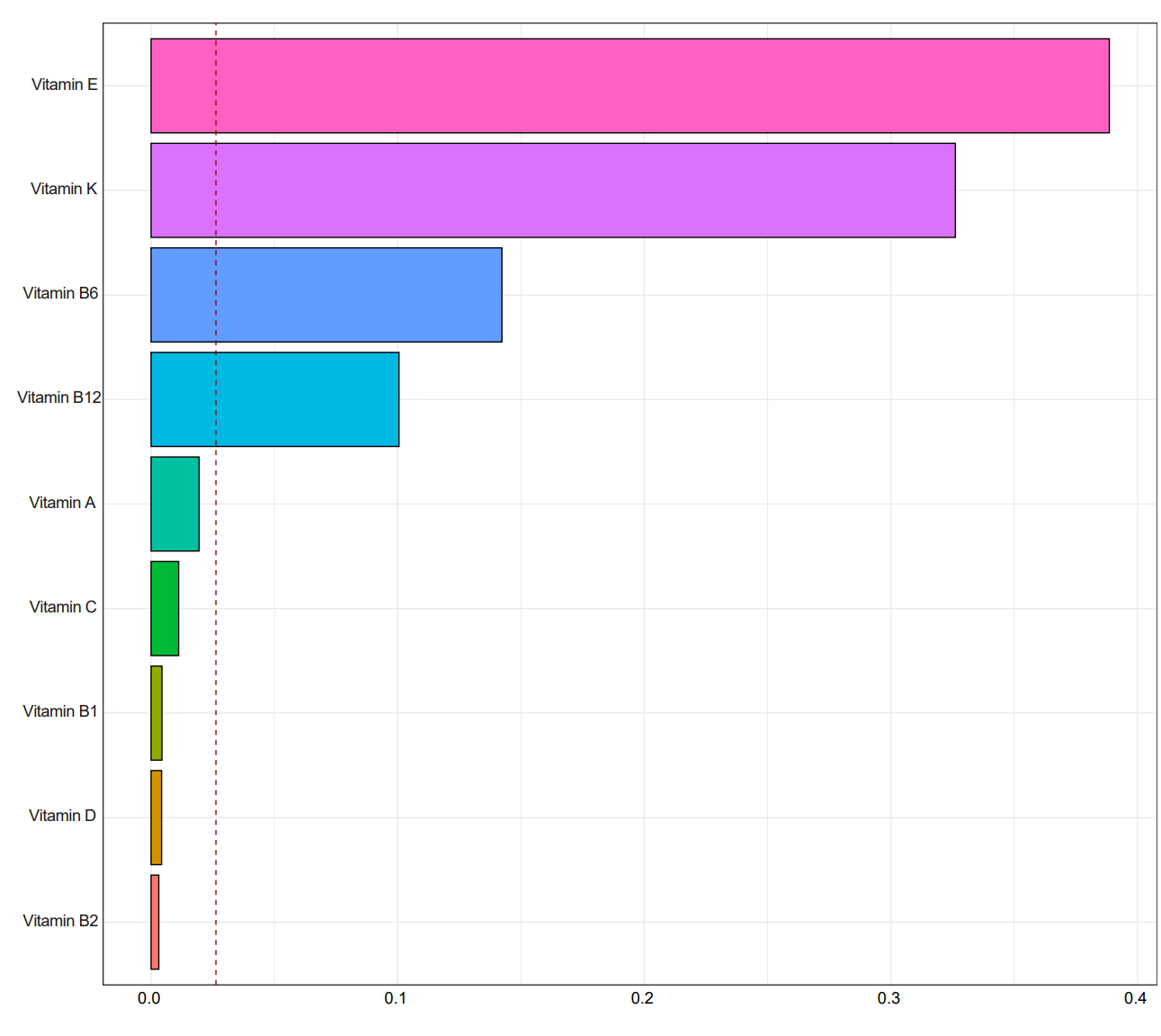


**Figure S3.** Visualization of weighted values for effect of dietary vitamin intake on CVD prevalence in weighted quantile sum models.

**Table S5.** The joint effect of nine dietary vitamins on CVD prevalence by WQS model in 2592 CKD subjects.

| Model | OR 95%CI | *P*-value |
| --- | --- | --- |
| WQS model | 0.77 (0.67, 0.88) | <0.001 |

Model was adjusted for age, sex, ethnicity, marital status, poverty-income ratio, education level, body mass index, smoking status, drinking status, diabetes, hyperlipidemia, and hypertension. Abbreviations: WQS, weighted quantile sum; OR, odds ratio; CI, confidence interval; CVD, cardiovascular disease; CKD, chronic kidney disease.

**
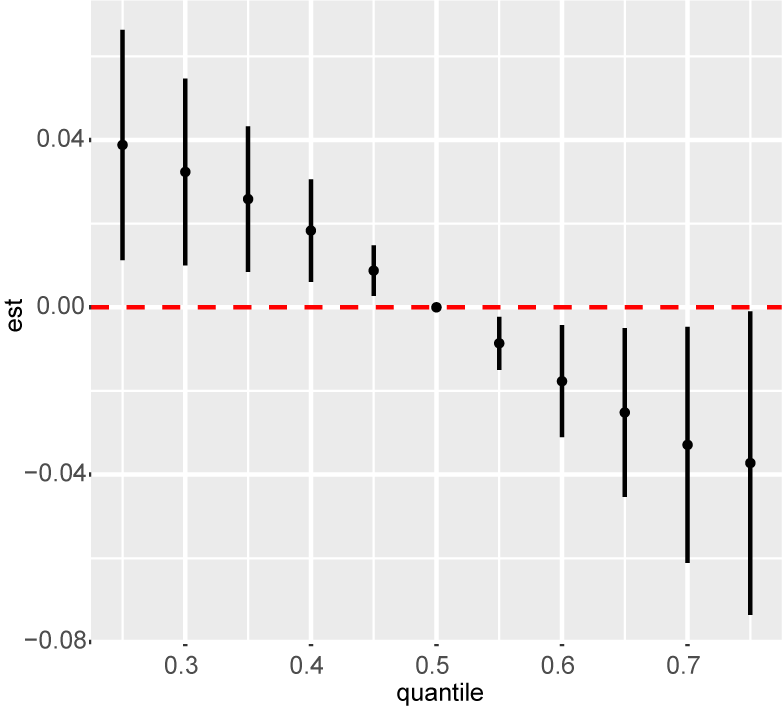
**

**Figure S4.** Combined effects of nine dietary vitamins mixtures and CVD incidence by BKMR analysis in 2592 CKD subjects. Model was adjusted for age, sex, ethnicity, marital status, poverty-income ratio, education level, body mass index, smoking status, drinking status, diabetes, hyperlipidemia, and hypertension. Abbreviations: CVD, cardiovascular disease; CKD, chronic kidney disease.
